# λ Phage Nanobioparticle Expressing Apoptin Efficiently Suppress Human Breast Carcinoma Tumor Growth *In Vivo*

**DOI:** 10.1371/journal.pone.0079907

**Published:** 2013-11-22

**Authors:** Alireza Shoae-Hassani, Peyman Keyhanvar, Alexander Marcus Seifalian, Seyed Abdolreza Mortazavi-Tabatabaei, Narmin Ghaderi, Khosro Issazadeh, Nour Amirmozafari, Javad Verdi

**Affiliations:** 1 Applied Cell Sciences Department, School of Advanced Technologies in Medicine, Tehran University of Medical Sciences, Tehran, Iran; 2 Research Center for Science and Technology in Medicine (RCSTiM), Tehran University of Medical Sciences (TUMS), Tehran, Iran; 3 Rajaie Cardiovascular, Medical and Research centre, Iran University of Medical Sciences, Tehran, Iran; 4 UCL Centre for Nanotechnology and Regenerative Medicine, Division of Surgery and Interventional Science, University College London, London, United Kingdom; 5 Medical Microbiology Department, School of Medicine, Iran University of Medical Sciences, Tehran, Iran; University of Chicago, United States of America

## Abstract

Using phages is a novel field of cancer therapy and phage nanobioparticles (NBPs) such as λ phage could be modified to deliver and express genetic cassettes into eukaryotic cells safely in contrast with animal viruses. Apoptin, a protein from chicken anemia virus (CAV) has the ability to specifically induce apoptosis only in carcinoma cells. We presented a safe method of breast tumor therapy via the apoptin expressing λ NBPs. Here, we constructed a λ ZAP-CMV-apoptin recombinant NBP and investigated the effectiveness of its apoptotic activity on BT-474, MDA-MB-361, SKBR-3, UACC-812 and ZR-75 cell lines that over-expressing her-2 marker. Apoptosis was evaluated via annexin-V fluorescent iso-thiocyanate/propidium iodide staining, flow-cytometric method and TUNEL assay. Transfection with NBPs carrying λ ZAP-CMV-apoptin significantly inhibited growth of all the breast carcinoma cell lines *in vitro*. Also nude mice model implanted BT-474 human breast tumor was successfully responded to the systemic and local injection of untargeted recombinant λ NBPs. The results presented here reveal important features of recombinant λ nanobioparticles to serve as safe delivery and expression platform for human cancer therapy.

## Introduction

Phages are a family of viruses that can only infect bacteria (there are 10^31^ of them) [1]. These nanobioparticles (NBPs) are extremely host specific and each type could infect specific species of a bacterium. Lambda (λ) is a temperate phage with a double stranded DNA genome. It's composed of glycoprotein E (gpE) and gpD coat proteins [2]. Lambda NBPs have high production capacity, high degree of stability, rapid and inexpensive production process and biological safety in human cells [3]. They are considered poor vehicles for transduction of eukaryotic cells [4] but the construction of phagemid vectors has made their manipulation easy [5]. Using phages is an attractive field of cancer therapy; but little is known about the bacteriophage mediated gene expression in eukaryotic cells. We therefore performed experiments to examine λ phage mediated apoptin expression both *in vitro* and *in vivo*.

Apoptosis is frequently occuring in many human tumor cells. It is also an important mechanism in chemotherapy for malignant cell death. Therefore, modulation of apoptosis by targeting pro-apoptotic and anti-apoptotic proteins is a powerful tool for treating cancers. It has been reported that an anti-cancer protein, apoptin, to induce the selective death of cell lines including melanoma, hepatoma, lymphoma, cholangiocarcinoma, colon carcinoma and lung cancer [6]–[8]. Apoptin was named because it was shown to induce apoptosis in tumorigenic human cells. Apoptin is a small protein of 121 amino acids (13 kDa) derived from chicken anemia virus (CAV) [9], [10]. CAV is one of the smallest avian viruses and does not grow in common cell lines. Its genome encodes three viral proteins (VP1-3) [11] that VP3 is also known as apoptin [10]. Its apoptotic activity is linked to its ability to localize in the nuclei of transforming cells, but not in the healthy human normal cells [12], [13].

Apoptin-mediated cell death is independent of death receptors such as FADD (Fas dependent death) or caspase-8, the key regulators of the extrinsic apoptotic pathway [14]. Phosphorylated Nur77 could transmit apoptotic signal from the nucleus to mitochondria, as it was shuttled from the nucleus to the cytoplasm upon transient expression of apoptin. Moreover, down-regulation of Nur77 protected against apoptin induced apoptosis [14]. Nur77 may cause *cytochrome c* release and activation of the apoptosome dependent death pathway. Apoptin is phosphorylated at threonine 108 in tumor cells [15]. This tumor-specific phosphorylation cause tumor-specific nuclear localization and apoptotic activity [15]. Apoptin involves caspase-3 that bypasses most of the upstream components of the apoptotic pathway [16]. Also it is influenced by regulators of the mitochondrial pathway like Apaf-1 that triggers *cytochrome c* release and activation of caspase-9 [17]. Apoptin interacts with the SH3 domain of p85, the regulatory component of phospho-inositide 3-kinase (PI3-K), through its proline-rich region [18]. Down-regulation of p85 cause nuclear exclusion of apoptin and impairs apoptosis, indicating that the interaction with the p85 is essential for cytotoxic activity of apoptin [18]. Over-expression of anti-apoptotic genes (*bcl-2*, *BAG-1* or *bcr-abl*) did not protect neoplastic cells from apoptin-induced apoptosis [17], [19], [20]. Also apoptin is independent of p53. Thus, apoptin is a potential therapeutic for the treatment of cancers, including those containing defects in any of the mentioned anti-apoptotic genes. Apoptin has advantages in contrast to conventional therapies that rely on the intact cellular apoptotic machinery such as chemotherapy or radiation.

Apoptin gene can be inserted into various vectors such as parvoviruses, papilomaviruses, polyomaviruses and adenoviruses [21], [22], making it attractive for cancer therapy. To use apoptin in cancer therapy, efficient delivery to tissues and proper expression of apoptin in neoplasms is required. Here we report the construction of a recombinant λ phage NBP expressing apoptin gene efficiently in breast cancer cell lines without targeting process.

Breast cancer is a heterogeneous carcinoma and thousands of genes may contribute to breast cancer pathophysiology, but subsets of tumors show the same patterns of genomic and biological abnormality [23]. So we tested the apoptotic effects of NBPs on BT-474, MDA-MB-361, SKBR-3, UACC-812 and ZR-75 that are *her-2* over-expressing cell lines. The *in vivo* studies were performed on BT-474 tumor bearing nude mice model.

Apoptin maintains its specificity for carcinoma cells when introduced and expressed by λ NBPs.

## Results

### Production of the recombinant λ NBPs

**Figure 1:**
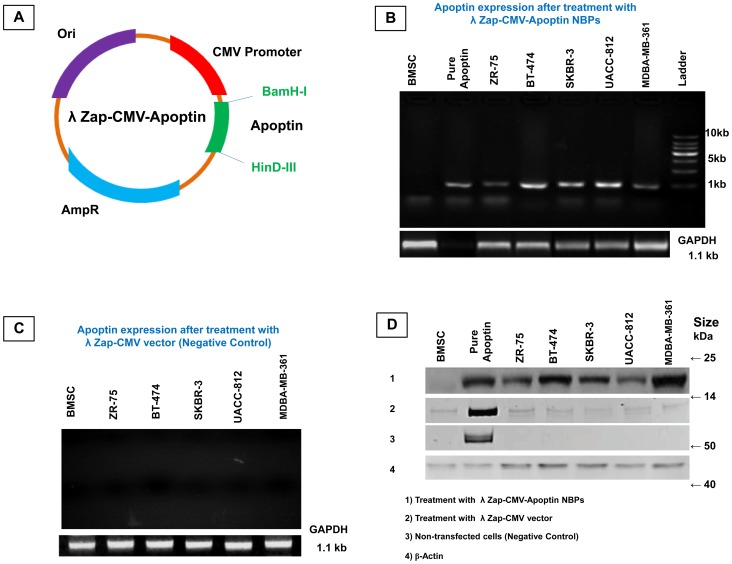
Design and Construction of NBPs harboring λ ZAP-CMV-apoptin expression plasmid. (**A**) The expression vector was subsequently inserted into the λ phage, generating the recombinant NBPs, (**B**) Different cell types were infected with the indicated recombinant λ NBPs at an MOI of 10 PFU/cell and apoptin transcription was analyzed by RT-PCR, (**C**) RT-PCR result for λ ZAP-CMV vector treated cells that have no expression of apoptin, (**D**) Western blot analysis to detect apoptin protein from cells supernatants and lysates. To analyze apoptin expression, the cells were infected with the indicated recombinant NBPs. Purified apoptin was used as a positive control and CAV infected BT-474 cell was used as a negative control.

To generate NBPs expressing apoptin, the recombinant plasmid λ ZAP-CMV containing the apoptin gene was constructed (Figure 1A). The restriction endonuclease digestion pattern showed that apoptin gene was inserted correctly into the vector. The recombinant plasmid λ ZAP-CMV-apoptin was packaged into NBPs and then transfected into various breast carcinoma cell lines. These cells were her-2 positive and were expressed proteins involved in signaling processes and in cell-cell interactions. MDA-MB-361 has the highest and ZR-75 has the lowest expression levels of her-2 marker (Unpublished data). The correct transcription and translation of apoptin was determined by reverse transcriptase polymerase chain reaction and western blot analysis. The apoptin transcripts (mRNA) were detected in all carcinoma cells harboring λ ZAP-CMV-apoptin. Bone marrow stem cell (BMSC) that was the only non-neoplastic cell did not express apoptin mRNA (Figure 1B, C). In the western blot analysis all the carcinoma cell lines expressed apoptin except the CAV infected BT-474 cell. Also BMSC as a negative control has not any expression of apoptin (Figure 1D).

### Anti neoplastic effect of NBPs

**Figure 2:**
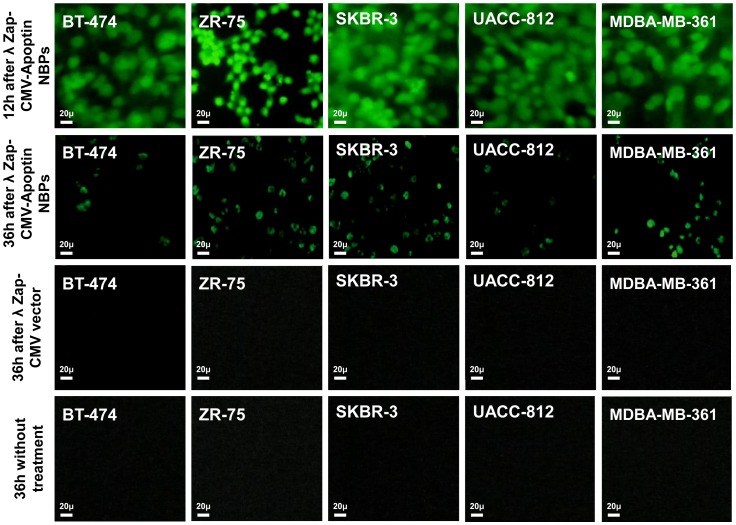
FITC immunostaining of apoptin expression in breast carcinoma cell lines. Immunostaining of apoptin protein showed that in can express in breast carcinoma cell lines after 12λ ZAP-CMV vector have not any apoptosis after 36 h as same as untreated cells.

**Figure 3:**
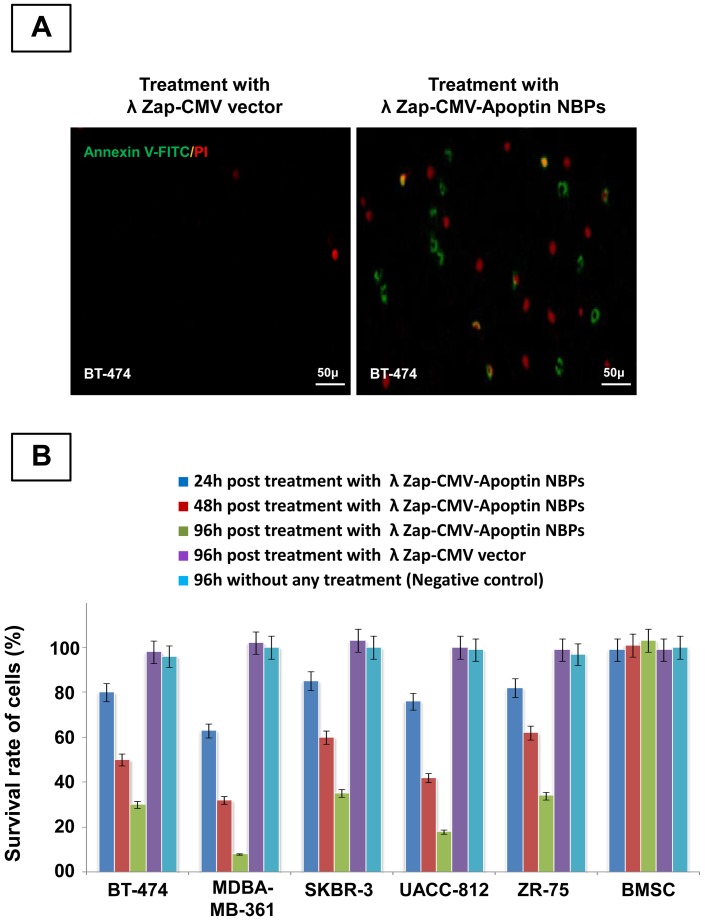
Cell viability and apoptosis after NBPs treatment. (**A**) Apoptosis in BT-474 breast carcinoma cell line induced by λ NBPs. The right side indicates NBPs treated group and Left side image indicates control group, (**B**) Cell viability determined by MTT dye reduction assay.

**Figure 4:**
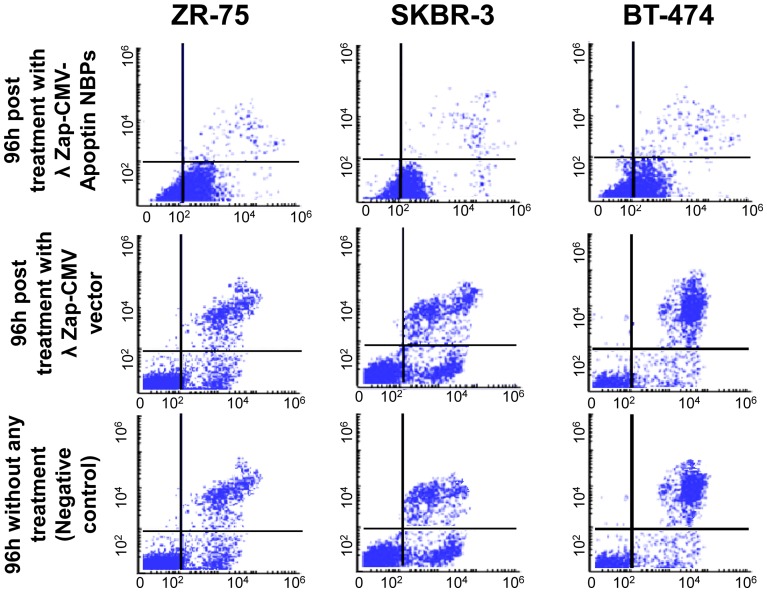
Confirmation of apoptosis by flowcytometry. BT-474, SKBR-3 and ZR-75 cells were examined after 36 h of transfection by flowcytometry. All the cell lines were susceptible to NBPs apoptin-induced apoptosis. We have not apoptosis in vector treated group and untreated group.

The anti neoplastic effect of λ NBPs was studied via morphological changes of cells using annexin-V fluorescein antibody and propidium Iodide (PI) and also flowcytometry for cultured cells *in vitro*. The immunofluorescent staining using the polyclonal antiserum in NBPs transfected cells showed expression of apoptin inside the nuclei of the transformed cells. NBPs transfected cells showed morphological changes of apoptosis (Figure 2). The expression of apoptin did not completely disappear after 36 h, but at this time between 40–70% of the cancer cells were dying. The study revealed clearly that apoptin was expressed by NBPs that harbors λ ZAP-CMV-apoptin cassette. No such changes were observed in λ ZAP-CMV vector treated groups. Also there was no expression of apoptin in BMSC treated NBPs (Figure 2). Annexin-V fluorescein/PI staining confirmed the apoptosis in the transformed cells. PI staining revealed color changes in BT-474 apoptotic cells (Figure 3A). Various lengths of transfections were compared for their effect on cell viability. The MTT colorimetric assay was performed to detect cell viability after transfection. When cells treated with λ NBPs the growth of the BT-474 cell line was inhibited by 20% after 24 h, 50% after 48 h and more than 70% after 96 h. As expected, with longer transfection times, the growth of the cells was inhibited (Figure 3B). The SKBR-3 cell line was inhibited by 15% after 24 h and about 65% after 96 h. The most inhibition of cell growth was seen in the MDA-MB-361 cell line by only 10% survival rate (Figure 3B). In contrast, the recombinant λ NBPs had no effect on BMSCs viability. There was no significant difference in the neoplastic cell viability after transfection with λ ZAP-CMV pure vector and non treated group as the negative control (Figure 3B). Also the viability of cells after NBPs treatment was investigated in three breast carcinoma cell lines by flowcytometry. As shown in the Figure 4, BT-474, SKBR-3 and ZR-75 cells were susceptible to NBPs-induced apoptosis. The λ ZAP-CMV vector lonely didn't affect cell viability and there was no change in the non-treated group. The flowcytometric assay confirmed the apoptosis results obtained from previous studies (Figure 4).

### Cytotoxicity of NBPs

**Figure 5:**
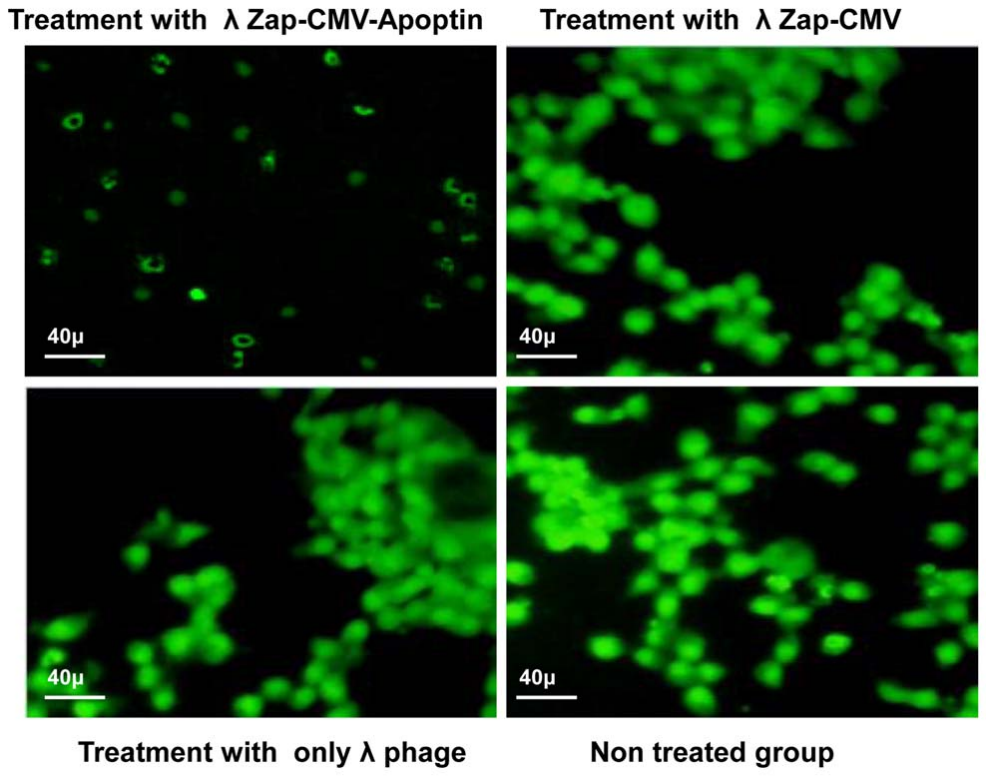
Cytotoxicity evaluation of plasmid and vehicle. BT-474 breast carcinoma cell line transfected with λ ZAP-CMV-apoptin, λ ZAP-CMV vector and λ phage (vehicle) construct stained with FITC immunostaining and then visualized by fluorescence microscopy. There was no sign of cell necrosis after the treatments. There is only apoptotic morphology of cells after treatment with λ ZAP-CMV-apoptin.

To determine the cytotoxicity of the λ ZAP-CMV-apoptin plasmid and only the λ phage they were transfected into BT-474 cells separately. The apoptosis of BT-474 cells was observed after transfection with of λ ZAP-CMV-apoptin plasmid (Figure 5), while in contrast λ ZAP-CMV plasmid and the only λ phage had no effect on the cell survival (Figure 5). Therefore λ NBPs not seems to exhibit cytotoxicity in cells and the apoptosis is induced by λ ZAP-CMV-apoptin construct due to expression of apoptin only in the malignant cell nucleus (Figure 5).

### Nude mice model for the study of recombinant NBPs effect

**Figure 6:**
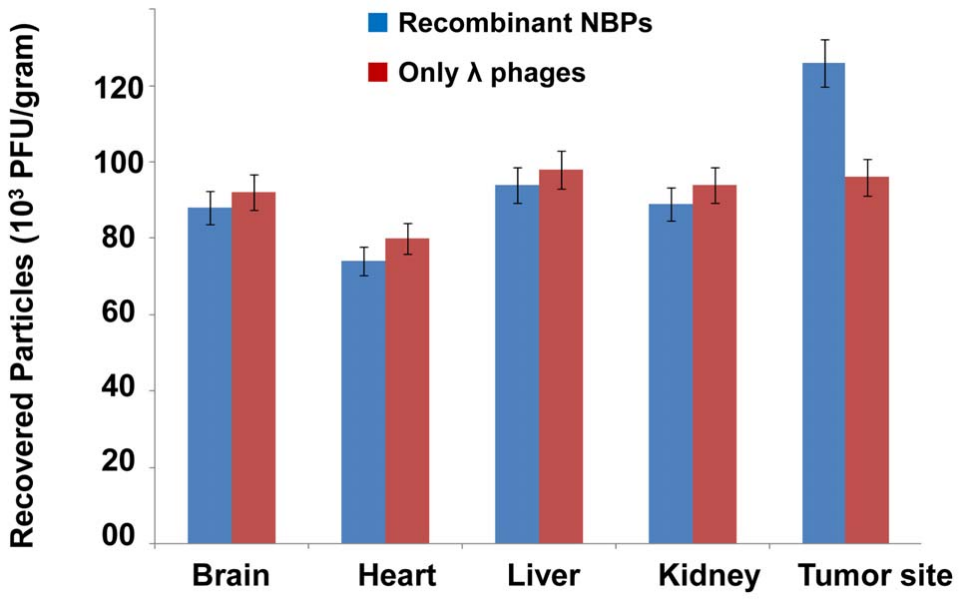
Recovery of NBPs from nude mice tissues. There were no significant differences in recombinant NBPs titer that was recovered from the different tissues of the treated mice. The tumor site was the only part of the mice body to support the more NBPs accumulation.

To investigate the ability of the recombinant NBPs *in vivo*, we injected NBPs into the tail vein (Intra Venous; IV) of mice bearing BT-474 human breast tumor and then recovered them after perfusion. The circulation time for NBPs is just 1 hour after IV injection. We determined the titers of the phage in tumors and normal organs as control sites (kidney, liver, brain and heart). NBPs showed not specific homing site (Figure 6) and recovered from all the mice organs even in the local intramuscular (IM) injected mice (after 6 hours), but surprisingly they were recovered from more percentage from the tumor site (Figure 6). The circulation time for NBPs is about 6 hours after IM injection.

**Figure 7:**
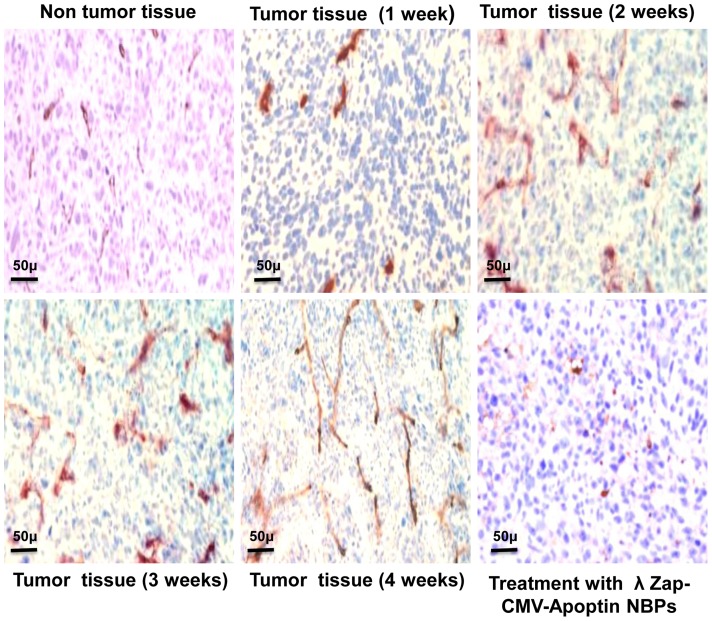
Evaluation of angiogenesis in tumor tissue. The nude mice were sacrificed and tumor samples collected at the end of the treatment period. Tissues were prepared and stained for CD34 as described in Materials and Methods. The tumor tissue develops angiogenesis. Amount of CD34 positive cells increases with a time.

**Figure 8:**
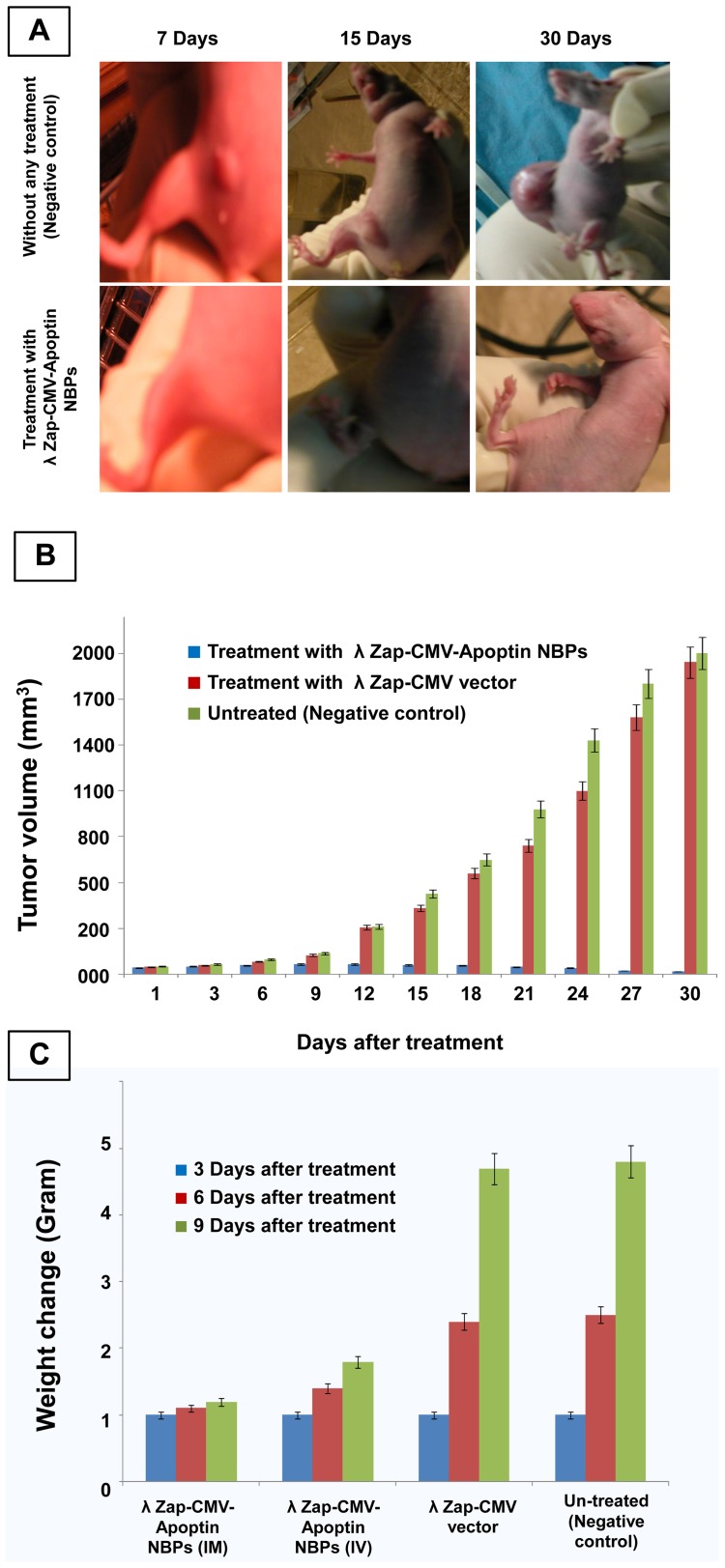
Determination of tumor size in nude mice. (**A**) Treatment of nude mice bearing human breast cancer xenografts with BT-474. Injection of BT-474 breast carcinoma cells to the nude mice resulted in the growth of xenografts. The Tumors were injected 3 times a week with 10^9^ PFU of recombinant NBPs, only vector and PBS. Hereafter, tumor growth was measured regularly, (**B**) Volumes of the BT-474 tumor tissue during treatment. Median tumor volume is determined over time in all treated groups. Tumor volumes were calculated using the equation: Length × width^2^ ×0.52, (**C**) The effects of different treatments on change in body weight over the treatment period. Average body weight was calculated for each treatment group at the indicated time points.

**Figure 9:**
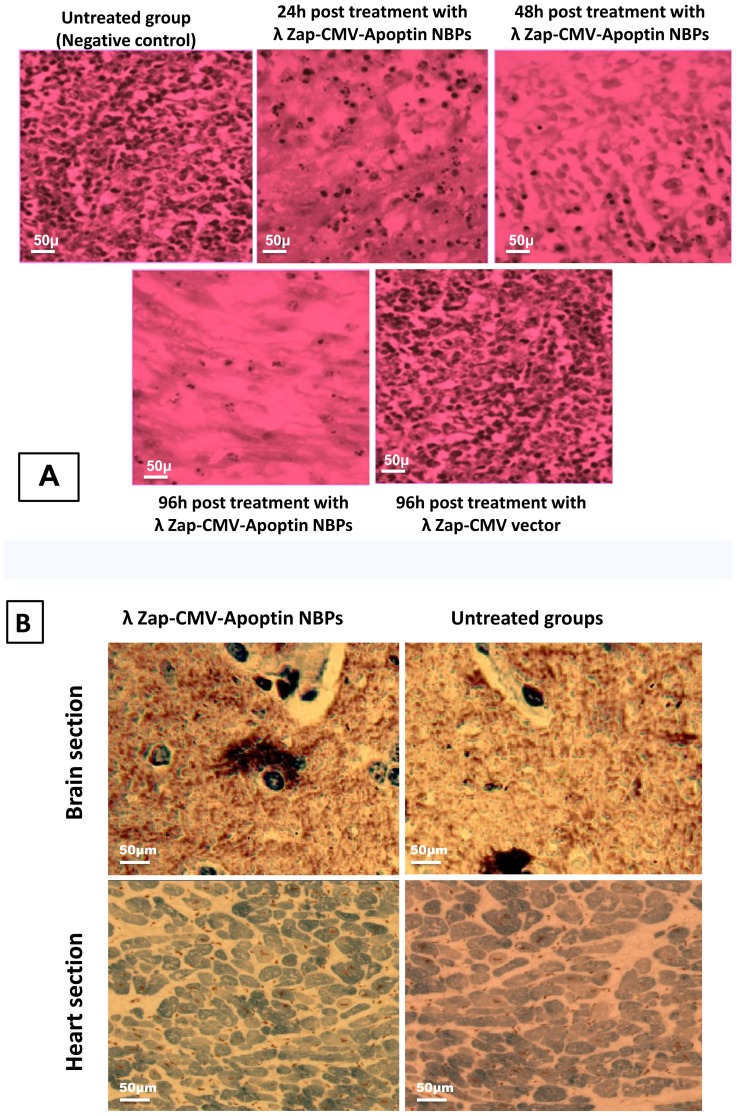
Immunohistochemical results of NBPs treated animals. (**A**) Histochemistry analysis of tumor tissue sections showing apoptotic changes. The untreated tumor tissue contains many dividing cells. After 96 h treatment with NBPs there are a few cells maintained in the tumor tissue that could proliferate. Tumor growth was markedly suppressed in the apoptin treated group, (**B**) Histological examination of other organs (brain and heart) in tumor bearing mice that is not involved in the pathological changes of BT-474 cells. There are no changes in morphology of the brain and/or heart tissues and they are as same as control groups.

**Figure 10:**
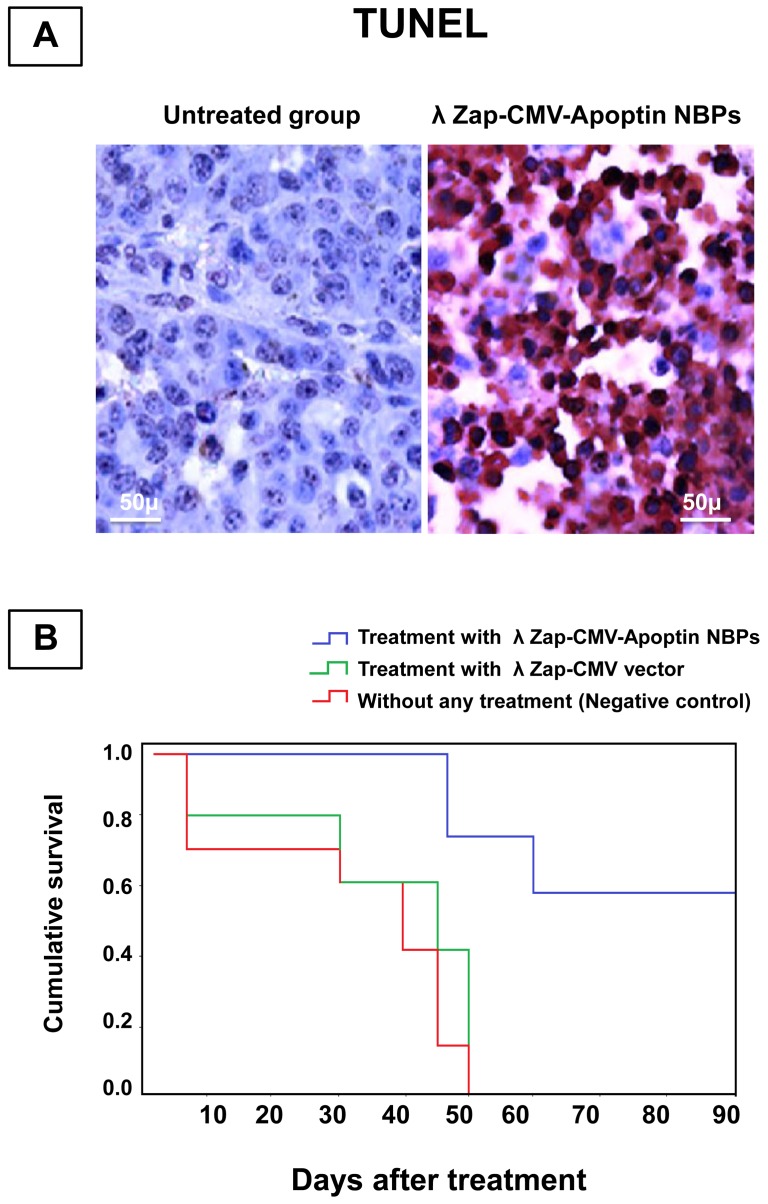
The survival rate of nude mice bearing human breast cancer. (**A**) TUNEL analysis revealed that apoptin induces the apoptotic activity in neoplasms (Magnification: ×400), (**B**) Analysis of survival. Mice treated with NBPs survived longer than the mice in the other 2 groups and the mean survival of NBPs-infected mice were >90 days (p<0.005). Fifty days after the beginning of the treatment, 90% of the animals infected by NBPs were alive, while at this time 100% of vector treated mice and 100% of saline-treated mice had died. Tumor-bearing mice treated with saline had a mean survival of 50 days.

Angiogenesis studies showed significantly higher expression of CD34 marker in immunohistochemical analysis that demonstrates neo-vascularization system in BT-474 tumors (Figure 7). CD34 is an indicator of vascularization density in tissues. After a time and enlargement in tumor size there is an increase in tissue vascularization (Figure 7). Treatment of animals with NBPs caused a decrease in the number of CD34 positive cells. Measuring the tumor size in animals showed that recombinant NBPs markedly inhibited the tumor growth (Figure 8A) but there were no changes in the control group to inhibit the progressive rate of effect to tumor growth (Figure 8A). Histology samples revealed that 100 μg of NBPs inhibited tumor growth completely and induced apoptosis in the tumor cells, but the same concentration of a control phage had no inhibitory effect (Figure 9A). The results were revealed that the NBPs have restricted neoplastic cells only in 96 h after treatment (Figure 9A). There was no effect of apoptosis in the brain and heart tissues of recombinant NBP treated mice in histochemistry assays (Figure 9B). To determine the apoptotic cells in the tumor tissues, also sections of the tumors were prepared for TUNEL assay. From the Figure 10A it is obvious that mice received recombinant NBPs contained many TUNEL positive cells in the tumor site, while in untreated tumors there were not TUNEL positive cells (Figure 10A). The tumors in mice that received recombinant NBPs were significantly smaller than those in the control groups (P≤0.05) (Figure 8B). The tumor sizes in the IM and IV injected groups were 4 and 2.8 times smaller than control groups, respectively (Figure 8C).

Finally, we compared the survival rates of tumor bearing nude mice after treatment with recombinant NBPs or control groups over a period of three months. All the tumor model animals in the untreated group died (survival rate 0%). Six of ten mice were survived even after three months in the NBPs treated group (survival rate 60%) and their tumor was suppressed completely (Figure 10B).

## Discussion

Efficacy and specificity is an important requirement for successful cancer therapy. Here, we discuss the *in vitro* and *in vivo* transfection of the human breast neoplastic cells with the untargeted recombinant λ nanobioparticles carrying λ ZAP-CMV-apoptin construct. These NBPs resulted in an effective induction of apoptosis selectively in tumor cells.

Selective centralization in cancer cells while sparing normal cells is an emerging field in recent years. Apoptin is a new anti-cancer tool with great potential to destroy only cancerous cells [12], [13], [27], [28]. However efficient systems are required to deliver apoptin to the cancer cells or express apoptin within these cells. We suggested λ nanobioparticles for this purpose. In our study, the construction and characterization of recombinant λ phage for gene delivery and expression in mammalian cells is reported.

In the 1940s, Bloch showed that phages could inhibit growth of tumors in animals [29]. These results were confirmed by Dabrowska et al who showed that phages inhibited growth of B16 tumors by blocking integrins necessary for metastasis [30]. It has been shown that bacteriophage-mediated DNA vaccination gives rise to antibody levels that are higher than those produced after vaccination with a commercially available recombinant protein vaccine [31]. Phage displaying human immunodeficiency virus (HIV) mimotopes (peptides that mimic the structure of an antigenic epitope), corresponding to epitopes from the C-terminus of glycoprotein 120 (gpl20) of HIV have also been used to elicit antiviral antibodies [32]. March et al took the approach of using unmodified lambda to deliver a genetically encoded hepatitis B surface antigen (HBsAg) into mice and they then monitored the immune response against the encoded antigen, which induced a high humoral response in mice [33]. These findings outline the versatility of phage-based vaccines. Of course our work is so different from all of these works. Our recombinant NBPs can cure the breast cancer affected region by inducing apoptosis in neoplasmic cells without transferring any antigen or mimicking the epitopes of cancer cell markers that are the targets of cancer therapy. Because the goal of our work was not to elicit the immune responses of the host (Humoral or cell mediated immunity responses). This makes our NBPs unique in cancer therapy.

As described, the filamentous phages have been used for gene delivery vectors in some cases but there are few reports about using lambda phages for this purpose. Recently we showed that lambda phage could express nanobody against her-2 positive breast carcinoma cells [34]. In this work the human breast carcinoma was stopped efficiently by λ NBPs but there was no apoptosis in normal cells (BMSCs). Also pure λ ZAP-CMV vector had not apoptotic effects on the neoplasmic cells (Figure 2). Our developed λ vector encoding apoptin gene can efficiently express the apoptin protein in the eukaryotic cells especially in carcinoma cells (Figure 1B,C,D). In 2007 scientists treated melanoma tumors with genetically engineered phages that were able to accumulate in tumors and express some genes [35]. λ NBPs delivered apoptin gene was expressed in a functional form in human breast carcinoma cell lines (Figure 3A). It is an unconventional approach for cancer gene therapy that is based on the tumor specific activity. Surprisingly the λ NBPs were not targeted for the carcinoma cells but there was no expression in control cells (Figure 1B,C,D). The reason correlates with the cellular localization of the apoptin. In normal cells, it resides dominantly in the cytoplasm, but in tumors, it localizes to the nucleus [12], [13]. Also the phage distribution was higher in the tumor site of our model (Figure 6). The first reason for this phenomenon should be the high metabolism and excess blood circulation in the tumor site. As our data shows tumor sizes in the IM and IV injected groups were 4 and 2.8 times smaller than control groups, respectively (Figure 8C). Probably in IM injection the recombinant NBPs circulate in the tumor site and enter the neoplasmic cells in this region more effectively. In IV injection the NBPs circulate in whole body and a significant amount of them distribute to the other sites. However a significant number of these particles accumulate in the tumor site. The second reason could be explained by the studies from Eriksson and colleagues [35]. They observed regression of tumors after treatment with phages with no specificity for the tumor [35]. They suggested that the anti-tumor activities are mediated by the host immune system through tumor infiltrating neutrophils. The recruitment of the immune system is highly dependent on the phage accumulation at the tumor site [35]. Since phages are regarded as foreign by the immune system, the inflammatory process initiates near the tumor that contains inflammatory proteins. The phenomenon could partly be explained by the “danger model” [36] which suggest that tumor trauma or infectious agents may result in regression of tumors. Also we found more results about binding untargeted phages in the cancer cells both *in vitro* and *in vivo*. There are some reports that demonstrate the phage attachment into the plasma membrane of lymphocytes [37], [38]. It has been demonstrated that some phages possess in their structure proteins containing a KGD motif, which is a ligand for the b3 integrins on eukaryotic cells [39]. The tumor selectivity of apoptin renders it suitable for systemic therapy, which in the case of λ phage delivery and expression; there is no need for specific targeting. So it would be cost effective and preferable pharmaceutical choice.

From the point that the breast cancer is a heterogeneous disease, the use of different cancer cells without considering their phenotypes is a common problem neglected in many cases. The cell lines in our panel were selected for their ability to express *her-2* and proliferate to form clinically relevant and invasive tumors in women.

As shown in Figure 3B, the viability of carcinoma cells treated with recombinant λ ZAP-CMV-apoptin NBP significantly has reduced, compared to BMSC (*P≤0.05). The MTT results showed that the growth of the BT-474 cell line was inhibited by 70% after 96h. As expected, with longer transfection times, the growth of the cells was inhibited more and more (Figure 3B). The SKBR-3 cell line was inhibited by 65% after 96h and the most inhibition of neoplastic cells was seen in the MDA-MB-361 by only 10% survival rate after 96 h (Figure 3B). Our expressed apoptin selectively caused apoptosis in the human BT-474, MDA-MB-361, SKBR-3, UACC-812 and ZR-75 cell lines. In contrast, normal BMSC was not done under apoptotic process and maintained their differentiation potential in multi lineage colony formation assays (Figure 2). These results that confirmed by flowcytometry (Figure 4) indicate that recombinant NBPs could induce significant anti-cancer effects than treatment with control groups (λ ZAP-CMV vector and untreated groups). Also NBPs didn't exert any cytotoxic effect on normal cells. The cytotoxicity of the plasmid and vector phage was not significant in treated cells (Figure 5).

There are no other reports about apoptin expression and its delivery by λ phages, but others have reported that cholangiocarcinoma cells can be transduced by adenovirus [40]. Also there is a report that has shown replication deficient adenovirus vectors expressing apoptin have significant antitumor effects against xenografted hepatoma, in some cases leading to complete regression [41]. There is another reason for higher accumulation of NBPs in tumor site rather than other tissues. The results of neo-vascularization confirmed the significantly higher expression of CD34 marker in BT-474 tumors compared to non tumor tissues (Figure 7). Also immunohistochemical result revealed that there is an increase in tumor tissue vascularization after enlargement in tumor size after time (Figure 7). Enlargement in tumor size due to a rapid proliferation gives rise to hypoxic areas inside the tumor, which demands increased nutrients and oxygen supply. We suggest that the extensive angiogenesis and neo-vascularization could trap the recombinant NBPs. Tumor vessels have a high proportion of proliferating endothelial cells, lack of pericytes and improper basement membrane formation leading to an enhanced permeability. Nanoparticles (in the size range of 10–400 nm), can extravasate and accumulate inside the interstitial space of tumor because endothelial pores have sizes varying from 10 to 1000 nm. In addition, lymphatic vessels are absent or non-functional in tumors which cause inefficient drainage from the tumor tissue. Nanoparticles entered into the tumor site are not removed efficiently and are thus retained in the tumor. This passive phenomenon has been called the “Enhanced Permeability and Retention (EPR) effect,” discovered by Matsumura and Maeda [42]–[44]. Selective accumulation of our NBPs then occurs by the EPR effect. The EPR effect is becoming the gold standard in cancer-targeting drug designing and for almost all solid tumors (with the exception of prostate cancer and/or pancreatic cancer) [43], [45]. Very high local concentrations of nanocarriers can be achieved at the tumor, for instance 10–50 folds higher than in normal tissue [46]. So EPR is one reason for accumulation of recombinant NBPs in the BT-474 tumor site.

Our *in vivo* studies in nude mice showed that the concentration of NBPs in the tumor tissue was more than other tissues (Figure 6). It's true that untargeted NBP construct recovered from all body organs of treated mice, but immunohistochemistry analysis has shown its apoptotic effect only in the tumor tissues and there was no change in other tissues after NBPs treatment (Figure 9A,B). We thought that histochemistry and TUNEL analyses are useful to study the changes of apoptosis in tumors. The morphology of condensed, spherical nuclear chromatin structures inside the cells reveals the apoptosis. Increase in NBPs accumulation in tumor tissues correlates with the increase in therapeutic efficacy. It decreased the tumor size (Figure 8A, B, C) and induced many TUNEL positive cells (Figure 10A) that decreased the mice death rate to 40% in comparison with control cases (Figure 10B). Finally further improvements in definition of the proper dose of the apoptin expressing NBPs could help to develop apoptin as an anti-cancer drug.

### Conclusion

This report describes the construction of a recombinant λ nanobioparticle that express apoptin and induce apoptosis only in transformed cells without targeting process. With this approach, we achieved in efficient regression of breast tumors with a combination of apoptin and λ nanobioparticles. Specificity of apoptin compensates targeting of phage vector and thus providing proof of principle that this agent can be used as a safe and inexpensive source of tumor therapy.

## Materials and Methods

### Strains and media

*Escherichia coli* DH5α (Pasture Institute, Tehran, Iran) was used as a host for λ phage amplification and titration purposes. The Luria-Bertani (LB) (Sigma-Aldrich, USA) and 2YT media (Invitrogen, USA) was supplemented with 100 μg/ml ampicillin (Sigma, USA) and kanamycin (Santa Cruz, USA) for the selection of transformants. The LB medium (supplemented with 0.01M MgCl_2_) and M9-medium (supplemented with ampicillin and isopropyl-β-D-1-thiogalactoside; IPTG) were used as electroporation and expression medium, respectively. Chicken anemia virus (CAV) that harbors apoptin gene was a gift from the Veterinary campus of Tehran University (Tehran, Iran).

### Cells and cell culture

The breast carcinoma cell lines (BT-474, MDA-MB-361, SKBR-3, UACC-812 and ZR-75) (Pasture Institute, Tehran, Iran) and normal mouse bone marrow stem cell (BMSC, Lonza Clonetics, USA) were grown in RPMI 1640 complete medium (Invitrogen, USA) and DMEM/F12 (Gibco, UK) containing 10% fetal bovine serum (FBS, Gibco, UK) at 37°C and 5% CO_2_. BMSC was used as a non-neoplastic cell that did not express her-2 marker as a negative control.

### PCR amplification of the apoptin gene

PCR amplification of the apoptin gene was carried out using the primer set that was designed based on the sequences in GenBank (NC-001427), and synthesized by Cinnagen Inc (Tehran, Iran). The first pair of designed primers was: (sense) 5′-ATGAATGAACGCTCTGCAGGAAGATACTCC-3′ and (antisense) 5′-CTTCAGTCTTATACGCCTTTTTGCGG-3′. The expected product size was 396 bp containing the complete apoptin open reading frame. The reaction mixture was prepared in PCR buffer containing 2 mM MgCl_2_, 200 mM dNTPs and 20 pmol of primers. Taq polymerase (1unit; Promega, USA) and template DNA (5 ng) from CAV were added to a total reaction volume of 25 μl. The reaction was carried out in an automated thermal cycler (Peq Lab, Germany) with an initial denaturation for three minutes followed by 35 cycles of denaturation at 95°C for one minute, annealing at 60°C for one minute and extension at 72°C for one minute and the final extension was carried out at 72°C for five minutes. A specific PCR-amplified product was purified from the gel by using a gel-extraction kit (QIAex II, Qiagen) and was used for cloning.

### Cloning of the apoptin gene

The PCR product gel-purified apoptin was cloned into the pCR2.1 vector (Invitrogen, USA). The plasmids were transformed into *E. coli* DH5α by electroporation. The apoptin cDNA cloned in pCR2.1 vector was confirmed by restriction enzyme digestion and by DNA sequencing. The insert was released from the pCR2.1 vector by digesting with *BamH-I* restriction enzyme and the released product was purified from the gel. It was ligated into *BamH-I* and *HinD-III* digested λ ZAP-CMV expression vector (Stratagene, USA) using 2U of T4 DNA ligase (Fermentas, USA) and 0.5 μl of 10 mM ATP (Invitrogen, USA). Then it was electroporated into electrocompetent DH5α and plated for separation of individual clones as described previously [24].

### Construction of recombinant λ NBPs

To generate the recombinant λ NBPs by homologous recombination; DH5α *E. coli* was infected with 1 multiplicity of infection (MOI) of λ nanobioparticles (Nanjing, China) and after 2 h incubation at 37°C, electroporated with 10 μg λ ZAP-CMV-apoptin. The ligated expression vector (λ ZAP-CMV-apoptin) was added to the packaging extract. The λ ZAP-CMV-apoptin tube was incubated at room temperature for 2 h. One ml of phage buffer [included 5.6 g NaCl, 2.0 g MgSO_4_,7H_2_O, 50.0 ml 1.0 M Tris-HCl (pH 7.5), 5.0 ml gelatin 2% (w/v) in 1 l distilled water] was added to the tube. Then, 20 μl of chloroform (Sigma, USA) was added and mixed the contents of the tube, gently. The tube was spined and the supernatant containing the NBPs was ready for titration. NBPs were titrated by plating the infected DH5α on agar plates (Gibco, USA) according to the manufacturer's instructions. Large scale NBPs containing λ ZAP-CMV-apoptin expressing vector was conducted in 1 l of M9 medium (Sigma-Aldrich, USA) containing ampicillin (Sigma, USA). After 24 h the culture was induced by adding 1 mM IPTG and was incubated a further 72 h. Induced culture was centrifuged at 8500 *g* for 30 min. The NBPs present in this extract was purified by immobilized metal affinity chromatography (Qiagen, USA). One clone was selected for high expression of the apoptin gene as determined by both western blot and reverse transcription-polymerase chain reaction (RT-PCR).

### In vitro expression studies

Human BT-474, MDA-MB-361,SKBR-3, UACC-812 and ZR-75 cell lines were cultured in RPMI-1640 (BT-474, SKBR-3, ZR-75) and DMEM complete medium (MDA-MB-361, UACC-812). The cells achieved 80% confluence at 24 h in the six-well culture plates. BMSC (without her-2 expression) was cultured in DMEM/F12 showing 80% confluency after 36 h. All the cultures were used for expression study of the λ ZAP-CMV-apoptin cloned vector. Serial phage dilutions ranging from ∼10^8^ M to 10^12^ M were prepared in calcium buffer (2 mM CaCl_2_, 150 mM NaCl, 10 mM HEPES, 3 mM NaN_3_, pH 7). The monolayer cells were harvested from plates and transferred into microtubes and then infected by recombinant NBPs containing λ ZAP-CMV-apoptin cassette. The contents of tubes were then mixed together and kept at room temperature for 15 min. The mixtures were cultured in the six-well plates in previously described media containing 5% FBS. After 4 h incubation, the medium over the cells was removed and fresh medium was added. At the end of 48 h, medium was removed completely; cells were used for RT-PCR, western blot and immunocytochemistry analyses.

### Identification of recombinant NBPs by RT-PCR

The transfected host cells were rinsed twice with phosphate buffer saline (PBS) and total RNA was isolated from cells using an RNeasy total RNA Extraction Kit (Qiagen, Germany) according to the manufacturer's instructions. One microgram of total RNA was reverse transcribed to first-strand cDNA with Superscript reverse transcriptase (Invitrogen, USA) as described in manufacturer description order. For the RT-PCR analysis, 5 μg of total RNA were reverse transcribed by using MMLV reverse transcriptase (Promega, USA). RNA equivalent cDNA (500 ng) was amplified by PCR using Taq DNA polymerase (Cinnagen, Iran) for 30 cycles under the following conditions: 95°C for 5 min, 55°C for 30 sec and 72°C for 1 min. β-actin was used as a housekeeping gene. PCR products were run on 1.5% agarose gel containing ethidium bromide and photographed using an imaging system.

### Western blot analysis for apoptin detection

After 36 h post transfection, recombinant λ NBP transfected cells were subjected to blotting analysis as described previously [25]. Briefly the cells were washed with phosphate buffer saline and then posed into 100 μl lysis buffer (50 mM Tris, pH 7.4, 250 mM NaCl, 5 mM EDTA, 0.1% Triton X-100, 20 mM β-glycerophosphate, 1 mM phenyl-methyl-sulphonyl-fluoride, 200 mg/ml trypsin inhibitor), transferred into a microtube and kept on ice for 30 min. Samples were boiled in sample buffer (50 mM Tris, pH 7.4, 2% SDS, 100 mM dithiothreitol, 0.1% bromophenol blue, 10% glycerol) for 5 min and cell debris was removed by centrifugation at 12000 *g* at 4°C for 10 min. Samples were loaded onto 10% SDS PAGE and blotted with mouse polyclonal anti-apoptin antibody for 2 h followed by incubation with goat anti-mouse IgG antibody (Santa Cruz, USA) labeled with HRP for 2 h. Pure apoptin was used as a positive control, CAV infected cells was used as a negative control and β-actin was used as internal control.

### Anti neoplastic effect of recombinant NBPs

The *in vitro* effect of apoptin in cells transfected by recombinant λ NBPs revealed as follows: Cells were plated on coverslips in dishes the day before transfection. Transfection was carried out using recombinant NBPs containing λ ZAP-CMV-apoptin. Twelve hours after transfection, and after that in 12 h interval cells were stained with FITC Annexin-V and propidium iodide (PI), mounted on slides, and observed by using a fluorescence microscope. Percent apoptosis were scored as the percent of 100 FITC fluorescence-positive cells showing apoptotic morphology.

### MTT colorimetric assay

The MTT (3-[4,5-dimethylthiazol-2-yl]-2,5-diphenyltetrazolium bromide, Sigma, USA) assay was performed to detect cell viability after recombinant NBPs transfection. The all kinds of host cells were seeded in 96-well plates (10^4^cells per well) one day before cells were transfected with recombinant NBP. Cell viability was measured every 12 h over a 96 h period by treating cells with 20 μl MTT (5 mg/ml) and incubating for 4 h at 37°C to allow MTT metabolization. The culture media was removed and the crystals formed was dissolved by adding 100 μl dimethylsulfoxide (DMSO, Merck, Germany) per well. The absorbance at 490 nm was measured with an enzyme-linked immunosorbent assay (ELISA) plate reader. Untreated cells were used as controls and all measurements were performed in triplicate.

### Flowcytometry assay

The changes in the membrane of apoptotic cells was determined by using an Apoptosis assay kit (Invitrogen, USA). The NBP expressing apoptin transfected BT-474, SKBR-3 and ZR-75 carcinoma cells were stained with Fluorscein annexin-V conjugate violet ratio metric asymmetry probe and the nuclei of the apoptotic cells were stained with red fluorescence. After staining, the cell suspensions were analyzed on a BD-flowcytometer. Viable cells were defined as annexin-V fluorescent iso-thiocyanate (FITC) negative events.

### NBP cytotoxicity assay

Cytotoxicity of λ ZAP-CMV-Apoptin NBPs, λ ZAP-CMV and only λ phage particles was tested by FITC fluorescence-positive cells showing apoptotic morphology. Transfected cells were placed on a 6-well plate, were washed with PBS and apoptotic cells that were differentiated by morphological changes visualized by fluorescence microscope.

### In vivo experiments on nude mice model

Thirty two nude mice were injected subcutaneously with BT-474 cells. The mice bearing breast cancer xenografts (∼2 cm^3^) divided into three study groups. One group was injected intravenous (IV) and intramuscular (IM) with 10^9^ PFU of the recombinant λ constructs and another group was taking control phage. The third study group of tumor bearing mice only injected with PBS. All the treatments were done for three days a week for 30 days. Mice body weights and tumor sizes were measured twice a week. Tumor volumes were calculated using the equation: Length × width^2^ ×0.52. In another study the xenograft tumors and other mouse organs (Brain and Heart) were removed and homogenized after perfusion. The NBPs bound to each tissue sample were recovered through the addition of host bacteria and titered on agar plates and expressed as PFU per gram of each tissue. Finally, the tumor tissues were prepared for histology analysis, detection of neo-vascularization and TUNEL (Tdt-mediated dUTP nick end labeling) assay. The samples were collected in 10% formalin and proper sections were made with a microtome. The sections were stained and studied for apoptotic changes under the light microscope. TUNEL was performed with the use of the *in situ* cell death detection kit (Roche, Mannheim, Germany), as described previously [26]. TUNEL staining was colored by means of 3-amino-9-ethylcarbozole. Next, by microscopic analysis the cells were examined for cell death induction. For detection of angiogenesis or neo-vascularization some sections of tumor tissues were then washed with PBS, incubated in blocking buffer with 5% bovine serum albumin for 20 min and probed for 60 minutes at room temperature with mouse anti CD34 antibody (Abcam, Inc., MA). Sections were then washed and sequentially incubated with a secondary antibody. For angiogenesis or neo-vascularization, CD34-labeled sections from three tumors per treatment group were visualized by light microscopy.

The handling of animals was in accordance with the Animal Care Guidelines' of Tehran University of Medical Sciences Ethical Committee. All the experiments were performed in the manners that minimize the stress and pain in animals.

### Statistical analysis

The statistical significance of differences was evaluated using the analysis of *t*-test with repeated measures using SPSS software (version 10.1). The survival rates of animals after 3 month follow up are shown in the Kaplan-Meier plot. All p-values reported were accepted as p<0.05.

## References

[1] Ackermann HW (2001) Frequency of morphological phage descriptions in the year 2000. Arch Virol 146(5): 843–857. 11448025 10.1007/s007050170120

[2] Murialdo H, Ray PN (1975) Model for arrangement of minor structural proteins in head of bacteriophage lambda. Nature 257: 815–817. 1186870 10.1038/257815a0

[3] Lankes H, Zanghi CN, Santos K, Capella C, Duke CM, et al (2007) *In vivo* gene delivery and expression by bacteriophage lambda vectors. J Appl Microbiol 102: 1337–1349. 17448169 10.1111/j.1365-2672.2006.03182.x PMC2063594

[4] Barbas CF, Burton DR, Scott JK, Silverman GJ (2001) Phage Display: A Laboratory Manual. New York: Cold Spring Harbor Press.

[5] Larocca D, Jensen-Pergakes K, Burg MA, Baird A (2001) Receptor targeted gene delivery using multivalent phagemid particles. Mol Ther 3: 476–484. 11319907 10.1006/mthe.2001.0284

[6] Leliveld SR, Zhang YH, Rohn JL, Noteborn M, Abrahams JP (2003) Apoptin induces tumor-specific apoptosis as a globular multimer. J Biol Chem 278: 9042–9051. 12496278 10.1074/jbc.M210803200

[7] Tavassoli M, Guelen L, Luxon BA, Gäken J (2005) Apoptin: specific killer of tumor cells? Apoptosis 10: 717–724. 16133863 10.1007/s10495-005-0930-3 PMC3533135

[8] Backendorf C, Visser AE, de Boer AG, Zimmerman R, Visser M, et al (2008) Apoptin: therapeutic potential of an early sensor of carcinogenic transformation. Annu Rev Pharmacol Toxicol 48: 143–169. 17848136 10.1146/annurev.pharmtox.48.121806.154910

[9] Noteborn MHM, de Boer GF, van Roozelaar DJ, Karreman C, Kranenburg O, et al (1991) Characterization of cloned chicken anemia virus DNA that contains all elements for the infectious replication cycle. J Virol 65: 3131–3139. 1851873 10.1128/jvi.65.6.3131-3139.1991 PMC240969

[10] Noteborn MHM, Koch G (1995) Chicken anemia virus infection: molecular basis of pathogenicity. Avian Pathol 24: 11–31. 18645763 10.1080/03079459508419046

[11] Meehan BM, Todd D, Creelan JL, Earle JAP, Hoey EM, et al (1992) Characterization of viral DNAs from cells infected with chicken anaemia agent: sequence analysis of the cloned replicative form and transfection capabilities of cloned genome fragments. Arch Virol 124: 301–319. 1605740 10.1007/BF01309811

[12] Danen-van Oorschot AAAM, Zhang YH, Leliveld SR, Rohn JL, Seelen MCMJ, et al (2003) Importance of nuclear localization of apoptin for tumor-specific induction of apoptosis. J Biol Chem 278: 27729–27736. 12754198 10.1074/jbc.M303114200

[13] Oro C, Jans DA (2004) The tumor specific pro-apoptotic factor apoptin (VP3) from chicken anaemia virus. Curr Drug Targets 5: 179–190. 15011951 10.2174/1389450043490631

[14] Maddika S, Booy EP, Johar D, Gibson SB, Ghavami S, et al (2005) Cancer-specific toxicity of apoptin is independent of death receptors but involves the loss of mitochondrial membrane potential and the release of mitochondrial cell-death mediators by a Nur77-dependent pathway. J Cell Sci 118: 4485–4493. 16179607 10.1242/jcs.02580

[15] Rohn JL, Zhang YH, Aalbers RI, Otto N, Den Hertog J, et al (2002) A tumor-specific kinase activity regulates the viral death protein Apoptin. J Biol Chem 277: 50820–50827. 12393903 10.1074/jbc.M208557200

[16] Danen-van Oorschot AAAM, van der Eb AJ, Noteborn M (2000) The chicken anemia virus-derived protein Apoptin requires activation of caspases for induction of apoptosis in human tumor cells. J Virol 74: 7072–7078. 10888647 10.1128/jvi.74.15.7072-7078.2000 PMC112225

[17] Burek M, Maddika S, Burek CJ, Daniel PT, Schulze-Osthoff K, et al (2006) Apoptin-induced cell death is modulated by Bcl-2 family members and is Apaf-1 dependent. Oncogene 25: 2213–2222. 16288204 10.1038/sj.onc.1209258 PMC2954965

[18] Maddika S, Wiechec E, Ande SR, Poon IK, Fischer U, et al (2008) Interaction with PI3-kinase contributes to the cytotoxic activity of Apoptin. Oncogene 27(21): 3060–3065. 18059340 10.1038/sj.onc.1210958 PMC2954967

[19] Danen-van Oorschot AA, den Hollander AI, Takayama S, Reed JC, van der Eb AJ, et al (1997) BAG-1 inhibits p53-induced but not apoptin-induced apoptosis. Apoptosis 2: 395–402. 14646536 10.1023/a:1026409808732

[20] Zhuang SM, Shvarts A, Jochemsen AG, van Oorschot AA, van der Eb AJ, et al (1995) Differential sensitivity to Ad5 E1B-21kD and Bcl-2 proteins of apoptin-induced versus p53-induced apoptosis. Carcinogenesis 16: 2939–2944. 8603467 10.1093/carcin/16.12.2939

[21] Olijslagers S, Dege AY, Dinsart C, Voorhoeve M, Rommelaere J, et al (2001) Potentiation of a recombinant oncolytic parvovirus by expression of Apoptin. Cancer Gene Ther 8: 958–965. 11781658 10.1038/sj.cgt.7700392

[22] Van der Eb MM, Pietersen AM, Speetjens FM, Kuppen PJ, van de Velde CJ, et al (2002) Gene therapy with apoptin induces regression of xenografted human hepatomas. Cancer Gene Ther 9: 53–61. 11924525 10.1038/sj.cgt.7700397

[23] Neve RM, Chin K, Fridlyand J, Yeh J, Baehner FL, et al (2006) A collection of breast cancer cell lines for the study of functionally distinct cancer subtypes. Cancer Cell 10: 515–527. 17157791 10.1016/j.ccr.2006.10.008 PMC2730521

[24] Sambrook J, Russel D (2001) Molecular cloning: a laboratory manual. 3rd Edition, Cold Spring Harbor, NewYork.

[25] Shoae-Hassani A, Amirmozafari N, Ghaemi A (2009) Virulence increasing of *Salmonella typhimurium* in Balb/c mice after heat-stress induction of phage shock protein A. Curr Microbiol. 59: 446–450. 10.1007/s00284-009-9458-z 19636618

[26] Peng DJ, Sun J, Wang YZ, Tian J, Zhang YH, et al (2007) Inhibition of hepatocarcinoma by systemic delivery of apoptin gene to the hepatic asiaglycorprotein receptor. Cancer Gene Ther 14: 66–73. 16874360 10.1038/sj.cgt.7700985

[27] Maddika S, Mendoza FJ, Hauff K, Zamzow CR, Paranjothy T, et al (2006) Cancer-selective therapy of the future: Apoptin and its mechanism of action. Cancer Biol Ther 5: 10–19. 16410718 10.4161/cbt.5.1.2400

[28] Gdynia G, Lehmann-Koch J, Sieber S, Tagscherer KE, Fassl A, et al (2008) BLOC1S2 interacts with the HIPPI protein and sensitizes NCH89 glioblastoma cells to apoptosis. Apoptosis 13: 437–447. 18188704 10.1007/s10495-007-0176-3

[29] Bloch H (1940) Experimental investigation of the relationship between bacteriophage and malignant tumors. Arch Gesamte Virusforsch 1: 481–496.

[30] Dabrowska K, Opolski A, Wietrzyk J, Switala-Jelen K, Boratynski J, et al (2004) Antitumor activity of bacteriophages in murine experimental cancer models caused possibly by inhibition of beta3 integrin signaling pathway. Acta Virol 48: 241–248. 15745047

[31] Clark JR, Bartley K, Jepson CD, Craik V, March JB (2011) Comparison of a bacteriophage-delivered DNA vaccine and a commercially available recombinant protein vaccine against Hepatitis B. FEMS. Immunol. Med Microbiol 61: 197–204. 10.1111/j.1574-695X.2010.00763.x 21204995

[32] Gomez-Roman VR, Cao C, Bai Y, Santamaria H, Acero G, et al (2002) Phage-displayed mimotopes recognizing a biologically active anti-HIV-1 gp120 murine monoclonal antibody. J Acqui Immune Defic Syndr 31(2): 147–153. 10.1097/00126334-200210010-00004 12394792

[33] March JB, Clark JR, Jepson CD (2004) Genetic immunization against hepatitis B using whole bacteriophage lambda particles. Vaccine 22: 1666–1671. 15068849 10.1016/j.vaccine.2003.10.047

[34] Shoae-Hassani A, Mortazavi-Tabatabaei SA, Sharif S, Madadi S, Rezaei-Khaligh H, et al (2013) Recombinant λ bacteriophage displaying nanobody towards third domain of HER-2 epitope inhibits proliferation of breast carcinoma SKBR-3 cell line. Arch Immunol Ther Exp 61: 75–83. 10.1007/s00005-012-0206-x 23224340

[35] Eriksson F, Culp WD, Massey R, Egevad L, Garland D, et al (2007) Tumor specific phage particles promote tumor regression in a mouse melanoma model. Cancer Immunol Immunother 56: 677–687. 16967280 10.1007/s00262-006-0227-6 PMC11031031

[36] Fuchs EJ, Matzinger P (1996) Is cancer dangerous to the immune system? Semin Immunol 8: 271–280. 8956455 10.1006/smim.1996.0035

[37] Kantoch M, Mordarski M (1958) Binding of bacterial viruses by cancer cells *in vitro* . Postepy Higieny i Medycyny Doswiadczalnej 12: 191–192. 13601395

[38] Wenger SL, Turner JH, Petricciani JC (1978) The cytogenic, proliferative and viability effects of four bacteriophages on human lymphocytes. In Vitro 14: 543–549. 680773 10.1007/BF02616097

[39] Gorski A, Dabrowska K, Switała-Jelen K, Nowaczyk M, Weber-Dabrowska B, et al (2003) New insights into the possible role of bacteriophages in host defence and disease. Medical Immunology 2: 2. 12625836 10.1186/1476-9433-2-2 PMC151275

[40] Pederson LC, Vickers SM, Buchsbaum DJ, Kancharla SR, Mayo MS, et al (1998) Combined cytosine deaminase expression, 5-fluorocytosine exposure, and radiotherapy increases cytotoxicity to cholangiocarcinoma cells. J Gastrointest Surg 2: 283–291. 9841986 10.1016/s1091-255x(98)80024-3

[41] Pietersen AM, Rutjes SA, van Tongeren J, Vogels R, Wesseling JG, et al (2004) The tumor-selective viral protein apoptin effectively kills human biliary tract cancer cells. J Mol Med 82: 56–63. 14647920 10.1007/s00109-003-0486-z

[42] Maeda H, Bharate GY, Daruwalla J (2009) Polymeric drugs for efficient tumor targeted drug delivery based on EPR-effect. Eur J Pharm Biopharm 71: 409–419. 19070661 10.1016/j.ejpb.2008.11.010

[43] Maeda H, Sawa T, Konno T (2001) Mechanism of tumor-targeted delivery of macromolecular drugs, including the EPR effect insolid tumor and clinical overview of the prototype polymeric drug SMANCS. J Control Release 74: 47–61. 11489482 10.1016/s0168-3659(01)00309-1

[44] Matsumura Y, Maeda H (1986) A new concept for macromolecular therapeutics in cancer chemotherapy: mechanism of tumori-tropic accumulation of proteins and the antitumor agent SMANCS. Cancer Res 46: 6387–6392. 2946403

[45] Unezaki S, Maruyama K, Hosoda J, Nagae I, Koyanagi Y, et al (1996) Direct measurement of extravasation of poly ethylene-glycol coated liposomes into solid tumor tissue by *in vivo* fluorescence. Int J Pharm 144: 11–17.

[46] Iyer AK, Khaled G, Fang J, Maeda H (2006) Exploiting the enhanced permeability and retention effect for tumor targeting. Drug Discov Today 11: 812–818. 16935749 10.1016/j.drudis.2006.07.005

